# Effects of Vaccination with Altered Peptide Ligand on Chronic Pain in Experimental Autoimmune Encephalomyelitis, an Animal Model of Multiple Sclerosis

**DOI:** 10.3389/fneur.2013.00168

**Published:** 2013-10-29

**Authors:** David H. Tian, Chamini J. Perera, Vasso Apostolopoulos, Gila Moalem-Taylor

**Affiliations:** ^1^School of Medical Sciences, University of New South Wales, Sydney, NSW, Australia; ^2^VA Consulting Services, Melbourne, VIC, Australia

**Keywords:** experimental autoimmune encephalomyelitis, altered peptide ligand, multiple sclerosis, mechanical allodynia, thermal hyperalgesia, nociception

## Abstract

Neuropathic pain is a chronic symptom of multiple sclerosis (MS) and affects nearly half of all MS sufferers. A key instigator of this pain is the pro-inflammatory response in MS. We investigated the behavioral effects of immunization with a mutant peptide of myelin basic protein (MBP), termed altered peptide ligand (APL), known to initiate immune deviation from a pro-inflammatory state to an anti-inflammatory response in experimental autoimmune encephalomyelitis (EAE), an animal model of MS. Male and female Lewis rats were injected with vehicle control or with varying doses of 50 or 100 μg guinea pig MBP in combination with or without APL. APL-treated animals established significantly lower disease severity compared to encephalitogenic MBP-treated animals. Animals with EAE developed mechanical, but not thermal pain hypersensitivity. Mechanical pain sensitivities were either improved or normalized during periods of clinical disease in male and female APL-treated animals as compared to the encephalitogenic group. No significant changes to thermal latency were observed upon co-immunization with APL. Together these data indicate that APL ameliorates disease states and selectively mediates an analgesic effect on EAE animals.

## Introduction

Multiple sclerosis (MS) is a chronic, T cell-mediated autoimmune neurological disease of the central nervous system (CNS) (1, 2), characterized by the production of acute multifocal CNS lesions with concurrent perivenular inflammation, demyelination, neuronal degeneration, and gliosis in gray and white matter (3, 4). While the cause of the disease is not known, pro-inflammatory CD4^+^ T cells, CD8^+^ T cells, B cells, macrophages, and natural killer cells have been implicated in disease onset and progression (4, 5). In addition, the dichotomous actions of pro- and anti-inflammatory cytokines are known to play a major role in disease exacerbation and amelioration respectively (6).

Neuropathic pain is a key clinical symptom in MS, with significant interference of quality of life (7–10). It results from damage to the nervous system (11), and presents in various forms such as ongoing extremity pain, paroxysmal neuropathic pain (e.g., trigeminal neuralgia, Lhermitte’s phenomenon), hyperalgesia (increased sensitivity to pain), and allodynia (pain produced by innocuous stimuli) (8, 12–14). While the mechanisms underlying MS-related neuropathic pain are not fully understood, lesions of CNS areas associated with pain, activation of T lymphocytes, and pro-inflammatory responses have been shown to contribute to the development and maintenance of neuropathic pain (15–17).

Experimental autoimmune encephalomyelitis (EAE) is a well-established and ubiquitous animal model that exhibits close clinical and histopathological similarities to various forms of MS (18). Following induction in susceptible animals by injections with a self-antigenic myelin-derived peptide, such as myelin basic protein (MBP), priming of antigen-specific lymphocytes occurs in the periphery. These cells subsequently migrate to the CNS, where they produce inflammatory mediators and cytokines that damage the myelin and axons and activate resident microglia to attract more inflammatory cells, resulting in inflammatory demyelination and neurodegeneration (19). Recent studies in rodents have demonstrated that animals with EAE develop neuropathic pain behaviors, including tactile and cold allodynia, and mechanical and thermal hyperalgesia (20–22). These symptoms have been observed before, during, and after neurological impairment, depending on the strain and the model used (17).

Epitopes derived from autoantigens involved in the autoimmune pathogenesis can be modified to modulate their immunological properties, and are called altered peptide ligands (APLs). APLs are similar to the immunogenic peptide, but with one or more amino acid substitutions in the essential contact positions, with the T cell receptor interfering with the T cell activation. Thus, APLs have the capacity to affect T cell receptor-mediated effector functions (23, 24), such as conferring an anergic effect on specific T cell subsets, or rendering them irresponsive to specific antigens despite presence of functioning antigen presenting cells (25). Different signaling mechanisms can also be activated to initiate a functional change in the T cell phenotype (24, 26), thereby altering cytokine production and downstream mechanisms (27) to selectively down-regulate pro-inflammatory T_H_1 cells (but not T_H_2 cells), as well as selectively inducing T lymphocytes that produce T_H_2 and T_H_0 cytokines (28). The ability for APLs to divert immune responses to a T_H_2 profile has been validated in several studies (26, 29–31). Importantly, T_H_2 cellular response has been shown to ameliorate EAE mediated by encephalitogenic T_H_1 population (28, 29, 31–33), as well as ameliorate pain states (15, 16, 34). As T_H_1 cells are widely believed to mediate pro-inflammatory effects, releasing a distinct set of cytokines that exacerbate pain states (35–37), their downregulation could similarly ameliorate clinical symptoms.

Our study builds upon the concept of immune deviation through APLs. While the effectiveness of myelin-derived APL in preventing EAE is well documented, we hypothesize that APL inoculation through immune deviation mediates an analgesic effect in this animal model of MS. Indeed, our results are the first to demonstrate that MBP-derived APL helps to restore EAE-affected mechanical pain thresholds.

## Materials and Methods

### Experimental animals

Male (*n* = 36) and female (*n* = 18) 6- to 8-week-old Lewis rats (Animal Resource Centre, Perth, WA, Australia) were used. Animals were housed with food and water *ad libitum* under 12-h light cycle, with constant humidity and temperature. Animals that developed mobility impairment were provided with soft jelly foods and easier access to water. Cage beddings changed twice a week, and animals were inspected daily for well-being. All animal experiments were approved by the Animal Care and Ethics Committee of the University of New South Wales, Sydney, NSW, Australia.

### Peptides

Encephalitogenic guinea pig MBP (gp-MBP) is known to induce acute T_H_1-associated EAE in Lewis rats (38). Doses of 50 or 100 μg gp-MBP (Sigma-Aldrich, NSW, Australia) were used to induce EAE. Previous studies using site-directed mutagenesis to compare different mutant peptides have shown that the peptide MBP_87–99_, with double Ala mutations at positions 91,96-[A^91^,A^96^]MBP_87–99_, alters immune responses leading to decrease in EAE severity (39–41). Our study was designed to assess the effects of this mutant peptide on sensory disturbances in EAE animals. In addition, cyclic peptides have been shown to be more stable *in vivo*, and have similar immunological activity to their linear counterparts (42). For increased stability, the [A^91^,A^96^]MBP_87–99_ peptide was cyclized from head to tail resulting in cyclo-(87–99)[A^91^,A^96^]MBP_87–99_. The cyclic double mutant cyclo-(87–99)[A^91^,A^96^]MBP_87–99_ peptide at a dose of 250 μg was used as the APL to inhibit EAE. Peptides were synthesized and purchased from Mimotopes Australia with purity greater than 95%.

### EAE induction

To induce EAE, rats were anesthetized (day 0) with 3% Isoflurane in oxygen, and inoculated at the base of the tail with a single subcutaneous injection of 200 μL inoculum. The control group was injected with an inoculum containing sterile saline (0.9% NaCl) emulsified with an equal volume of incomplete Freund’s adjuvant (IFA; Difco Laboratories, Detroit, MI, USA) supplemented with desiccated 1 mg/mL *Mycobacterium tuberculosis* (strain H37RA, Difco Laboratories). The IFA with *Mycobacterium tuberculosis* is defined as CFA. A second group was injected with either 50 or 100 μg gp-MBP emulsified in CFA (deemed the MBP group), while a third group was injected with equal dose of gp-MBP, but an additional 250 μg cyclo-(87–99)[A^91^,A^96^]MBP_87–99_ (APL group).

### Clinical assessment

Following disease induction, animals were assessed daily for signs of disease for up to 35 days, graded using the following scale: Grade 0, normal rat; Grade 1, flaccid tail; Grade 2, weak hind limbs with ataxia; Grade 3, hind limb paralysis; Grade 4, paraplegia with forelimb paralysis; Grade 5, moribund. Weight changes were also recorded.

### Pain assessment

Behavioral tests were conducted three times a week prior to and up to 4 weeks following EAE induction.

Prior to testing, animals were acclimatized in a clear Perspex 20 cm × 20 cm box standing 20 cm above the bench for 15 min until they were in a non-agitated state. Thermal hyperalgesia was assessed by exposing the plantar hind paw of the animal to radiant heat through the transparent floor of the Perspex box, using a plantar analgesia meter (Ugo Basile, Varese, Italy). A cut-off of 20 s was applied to prevent tissue damage.

Mechanical allodynia was then assessed subsequent to 30-min break, by an electronic von Frey anesthesiometer (Ugo Basile). The animal, placed upon an elevated wire mesh surrounded by a Perspex box, was exposed to increasing mechanical pressure to the plantar hind paw through a metal filament.

Withdrawal latency and threshold was measured automatically from the initiation of heat or mechanical stimulus to withdrawal of the paw, defined as sudden jerk of paw away from stimulus. This was repeated three times in both left and right hind paw separated by 2–5 min between each stimulus. Mean results for each animal was calculated.

### Data analysis

**Figure 1:**
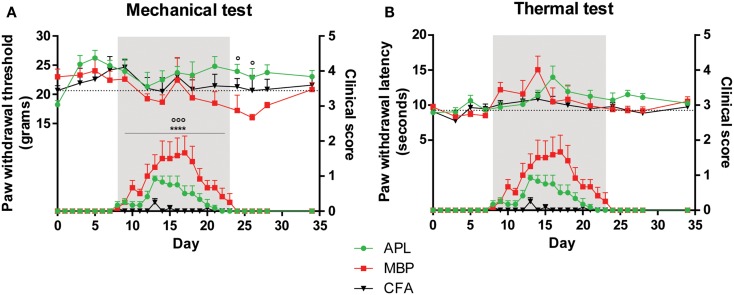
**Pain hypersensitivity (top set) and clinical score (bottom set) in male Lewis rats injected with 100 μg gp-MBP on day 0**. Top series represent response to stimuli, while bottom series represent clinical score. EAE clinical scores of rats immunized with MBP were significantly greater than those of APL-treated or CFA-immunized (control) animals during period of established disease. **(A)** MBP-treated animals demonstrated a decrease in paw withdrawal thresholds to mechanical stimulus, while APL-treated animals had significantly elevated mechanical pain thresholds, in particular during disease recovery. **(B)** No significant difference was observed in paw withdrawal latency to thermal stimuli between APL-treated and MBP-treated animals. (*n* = 6 per group; ****p < 0.0001, MBP compared to control; ^∘^*p* < 0.05, ^∘∘∘^*p* < 0.005, APL compared to MBP; two-way ANOVA with Bonferroni’s post-tests for upper panel, Mann–Whitney test for lower panel). Gray region indicates periods of established disease. Dotted line represents mean baseline values. Data expressed as mean ± SEM.

**Figure 2:**
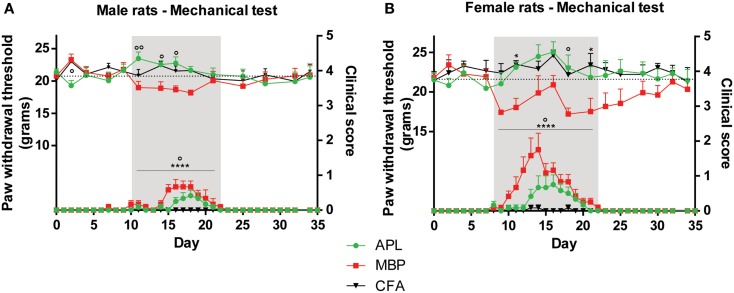
**Changes in mechanical sensitivity (top set) in male and female Lewis rats inoculated with 50 μg gp-MBP on day 0 compared to clinical scores (bottom set)**. In both males and females, EAE clinical scores of rats immunized with MBP were significantly greater than those of APL-treated or CFA-immunized (control) animals during period of established disease. **(A)** In males, APL conferred significant increase in mechanical threshold compared to MBP-treated rats on day 11, 14, 16. **(B)** In females, MBP animals generally exhibited lower pain thresholds, while APL animals maintained thresholds similar to control. (*n* = 6 per group; *p < 0.05, ****p < 0.0001, MBP compared to control; ^∘^*p* < 0.05, ^∘∘^*p* < 0.01, APL compared to MBP; two-way ANOVA with Bonferroni’s post-tests for upper panel, Mann–Whitney test for lower panel). Gray region indicates periods of established disease. Dotted line represents mean baseline values. Data expressed as mean ± SEM.

**Figure 3:**
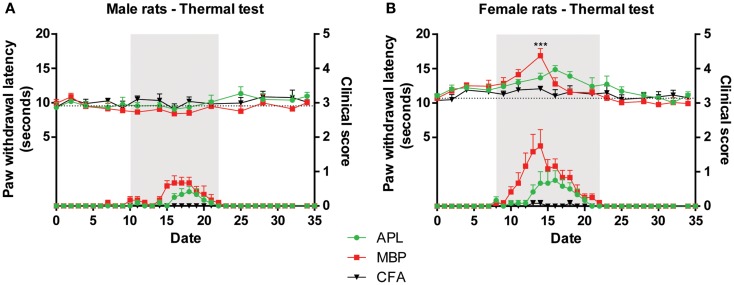
**Changes in thermal sensitivity (top) in male and female Lewis rats inoculated with 50 μg gp-MBP on day 0 compared to clinical scores (bottom)**. EAE clinical disease is as in Figure 2. **(A)** In males, no significant difference was observed between control, MBP-treated, and APL-treated animal groups. **(B)** In females, MBP evoked a transient increase in latency on day 14, with no other significant difference observed at any other time-points. (*n* = 6 rats per group, ***p < 0.001, MBP compared to control; two-way ANOVA with Bonferroni’s post-tests; upper panel). Gray region indicates periods of established disease. Dotted line represents mean baseline values. Data expressed as mean ± SEM.

In mechanical and thermal tests, raw scores for left and right hind paws were combined and averaged for each time-point. Time periods were classified as pre-disease, disease peak, and recovery, defined as time-points prior to development of clinical signs, presence of clinical signs, and resolution of clinical signs respectively. Disease peak periods are shaded gray in Figures 1–3.

For measurements of pain behaviors, data were analyzed with repeated measures two-way ANOVA with Bonferroni’s post-test, and for EAE clinical scores data were analyzed with a non-parametric Mann–Whitney test as appropriate, using GraphPad Prism v5.04 (GraphPad Software, Inc., CA, USA). Significance was set at *p* < 0.05. All data are presented as mean ± SEM.

## Results

### Effects of co-immunization with APL, cyclo-(87–99)[A^91^,A^96^]MBP_87–99_, in male lewis rats injected with 100 μg gp-MBP

We first studied the effects of APL vaccination on EAE clinical disease course and changes to mechanical threshold and thermal latency for pain in 18 male Lewis rats immunized with 100 μg gp-MBP. Three study groups were used, (i) animals injected with CFA only (vehicle control), (ii) animals injected with CFA + 100 μg gp-MBP (MBP group) and, (iii) animals co-immunized with CFA + 100 μg gp-MBP + 250 μg cyclo-(87–99)[A^91^,A^96^]MBP_87–99_ (APL group).

#### Treatment with APL significantly reduced disease severity in male animals with EAE

Both MBP-treated and APL-treated animal groups demonstrated characteristic clinical deficits beginning on day 8 (Figures 1A,B). Vehicle-treated animals displayed minimal clinical deficits. Onset was characterized by weakness of tail, followed by ascending motor deficits.

Disease severity in MBP-treated animals peaked later on day 17 with a mean maximal score of 1.7 ± 0.5 (*n* = 6), while the APL cohort peaked earlier on day 13 with 82% comparative reduction in average maximal severity (peak mean score 0.9 ± 0.08). APL animals recovered significantly earlier compared to MBP animals (22.5 days for MBP and 18.8 days for APL, Mann–Whitney test, *p* < 0.01).

Overall, MBP animals demonstrated significantly greater EAE clinical scores than vehicle-treated animals when clinical signs were present between day 8 and 23 (*p* < 0.0001, Mann–Whitney test). Compared to MBP, the APL group showed a significant reduction in clinical scores during the same period (*p* < 0.005, Mann–Whitney test).

#### Treatment with APL significantly increased thresholds to mechanical stimuli following disease resolution

Prior to disease onset on day 8 (Figure 1A) and during disease establishment (day 8–23, gray region), no significant differences in paw withdrawal thresholds to mechanical stimuli were observed between CFA-injected control, MBP-, and APL-treated animals (*p* > 0.05, two-way ANOVA). Following resolution of clinical disease, MBP animals developed reduced mechanical thresholds, while APL animals exhibited significantly elevated thresholds of as much as 6.90 g compared to MBP animals on days 24 and 26 (*p* < 0.05, two-way ANOVA). Although not statistically significant, compared to control animals, MBP animals generally possessed lower thresholds to mechanical pain, particularly after disease peak, whereas APL animals demonstrated elevated thresholds.

Interestingly, a sharp increase in withdrawal threshold was observed in MBP animals on day 16, corresponding closely to the rats’ maximal disease severity (score 1.7). While hindlimb paralysis has the potential to confound results, results for all three groups became relatively stabilized following animal recovery.

#### No significant changes to thermal latency were observed in animals co-immunized with APL

In addition to evaluating alterations of mechanical pain thresholds, we examined changes to paw withdrawal latency in response to thermal stimulation (Figure 1B). Interestingly, no difference was observed between any of the cohorts prior to, during, and following disease establishment (*p* > 0.05, two-way ANOVA). We conclude that male animals with EAE do not develop thermal pain hypersensitivity and that APL does not confer any significant effects on latency to thermal stimuli.

### Effects of co-immunization with APL, cyclo-(87–99)[A^91^,A^96^]MBP_87–99_, in male and female rats injected with 50 μg gp-MBP

To reduce confounding effects of severe hind limb paralysis on the ability of animals to withdraw their paw from the stimulus, we halved the MBP dose to 50 μg in order to minimize physical paralysis. Since previous studies have demonstrated differences between sexes in EAE severity and nociception, we tested both male and female rats for clinical EAE and pain sensitivity. Here we used 18 male and 18 female Lewis rats immunized with CFA only (control), CFA + 50 μg gp-MBP (MBP group), and CFA + 50 μg gp-MBP + 250 μg cyclo-(87–99)[A^91^,A^96^]MBP_87–99_ (APL group).

#### Treatment with APL reduced disease severity in both male and female animals with EAE

Disease onset in male MBP animals occurred around day 10 and in female MBP animals around day 8 (Figures 2A,B). Additionally, the disease course of male animals peaked later (day 16) than female animals (day 14). Recovery for both sexes occurred on day 23.

Significant differences between the disease course of male and female MBP animals were observed. Female animals developed clinical signs that were on average 151% more severe than male animals (result reported as percentage of difference compared to male animals, *p* < 0.05; Mann–Whitney test). For example, three female MBP animals developed scores of 3 (complete paralysis of tail and hind limbs), whilst the male MBP cohorts only managed to develop maximal scores of 1.5 (paralysis of tail only).

Separately, there existed a significant difference in clinical scores between CFA and MBP, and MBP and APL groups in both sexes when clinical signs were present (day 10–22 for males, day 8–22 for females) (Figures 2A,B). Both male and female MBP animals displayed significantly greater EAE clinical scores than CFA animals (*p* < 0.0001 in both, Mann–Whitney test). Both male and female APL animals showed a significant reduction in EAE severity as compared to MBP animals (*p* < 0.05, Mann–Whitney test) during established disease. These results highlight APL’s ameliorative effect on disease status in both males and females.

#### Treatment with APL significantly ameliorated mechanical pain sensitivities in both male and female animals during established disease

In male animals, APL normalized changes in EAE-induced mechanical pain thresholds only during periods when clinical signs were present. No significant difference was observed between the three cohorts prior to and following disease establishment, except a small reduction in threshold in APL-treated animals compared to MBP animals on day 2 (*p* < 0.05, two-way ANOVA). When clinical signs of disease were evident on day 10–22 (Figure 2A, gray region), MBP animals exhibited lower mechanical pain thresholds compared to control, while no significant differences were observed between APL and control (*p* > 0.05, two-way ANOVA). Furthermore, on day 11, 14, and 16, animals co-immunized with APL demonstrated threshold increase of at least 2.6 g against MBP animals (*p* < 0.05, two-way ANOVA) and their paw withdrawal threshold to mechanical stimulus was similar to control animals. This validates the ability for APL to ameliorate changes to mechanical pain thresholds in animals with EAE.

In female animals, APL’s ameliorative effects were similarly evident mostly during established disease (Figure 2B, gray region). Prior to disease onset, no significant differences in mechanical threshold were observed between the cohorts (*p* > 0.05, two-way ANOVA). MBP animals developed a sharp decrease in mechanical threshold starting on day 9 following disease induction, followed by a steady increase, potentially associated with the escalating disease severity. The threshold started to reduce again on day 18, following amelioration of paralytic signs. When clinical signs were present between day 8 and 22, MBP animals exhibited a general reduction in threshold, particularly noticeable on day 11 and 21 (*p* < 0.05, two-way ANOVA). During the same period, APL animals displayed similar thresholds to control, which were significantly elevated against MBP on day 18 (*p* < 0.05, two-way ANOVA). Again, this indicates the normalization of mechanical thresholds in APL animals with EAE.

#### No significant changes to thermal latency were observed in APL-treated animals

In male animals, no significant latency variations to thermal stimuli were observed between any time-points during the course of the experiment (Figure 3A; *p* > 0.05, two-way ANOVA), similar to the results from male animals inoculated with twice the MBP dosage (Figure 1).

In female animals, immunization with only MBP was able to increase thermal latency by 4.8 s on day 14 compared to control (Figure 3B; *p* < 0.001, two-way ANOVA). However, it should be noted that this transient increase occurred concurrent with disease peak, therefore it is possible that animal paralysis has confounded results. At the same time-point, APL animals, which exhibited less severe paralysis, displayed no significant difference compared to control. No other changes were observed during the course of the experiment.

## Discussion

Over the last two decades, numerous studies in rodents have demonstrated that several non-encephalitogenic myelin-derived APLs confer protection from the development of EAE, and even reverse established paralytic disease (26, 28, 29). These APLs were shown to induce T cells that are cross-reactive with the native myelin peptide, but modify the immune response and prevent autoimmune encephalomyelitis. Our results here have shown that active immunization with the APL cyclo-(87–99)[A^91^,A^96^]MBP_87–99_ in an animal model of EAE, not only mitigated the disease course, but also improved symptoms of mechanical pain hypersensitivity.

We demonstrated that in both male and female Lewis rats, co-immunization with gp-MBP and APL cyclo-(87–99)[A^91^,A^96^]MBP_87–99_ has significantly reduced EAE disease severity and shortened the disease course as compared to gp-MBP alone. These results are in line with previous studies (26, 28, 31, 43, 44). We also demonstrated that EAE severity is dependent upon MBP dosage and gender. It is well known that many autoimmune diseases, including MS, are more predisposed toward females than males (45). Our study determined female animals were significantly more affected by equal dosages of MBP, with clinical scores of female rats more than twice that of male animals. A dose of 100 μg MBP was able to elicit mean peak disease score of 1.7 in male Lewis rats, while 50 μg MBP was only able to generate considerably lower mean peak score of 0.7. Female rats were more affected by MBP immunization, with only 50 μg MBP producing a mean peak score of 1.75, comparable to doubling the dosage in male animals. Indeed, it has been shown that immunization with gp-MBP induced disease in all female mice, but only in half of male animals (46). Variations in encephalitogenic peptides and animal strains similarly indicated that female animals were more severely affected (20, 47, 48).

Chronic neuropathic pain arises subsequent to lesion or disease of the somatosensory nervous system (11). Recent studies have shown that T lymphocytes and pro-inflammatory cytokines play a significant role in the development and maintenance of neuropathic pain (34, 49). For example, injection of a T_H_1 cell population producing pro-inflammatory cytokines increased the level of neuropathic pain, whereas injection of a T_H_2 cell population producing anti-inflammatory cytokines attenuated pain sensitivity in nerve-injured rats (15). As chronic neuropathic pain affects the majority of MS patients (10, 17), it is believed that modulation of pro-inflammatory T cells and their associated cytokine response will mitigate such symptoms.

Our results show that concurrent inoculation with APL, in addition to disease-causing MBP, normalizes disturbances to mechanical pain threshold, particularly during established disease, although it had no significant effect on thermal latency to pain. Consistent with previous studies (21, 22, 50, 51), we found that animals with EAE display mechanical allodynia during the course of the disease. In addition, we observed normalization or increase in pain threshold to mechanical stimuli during periods of clinical disease in animals co-immunized with APL. While there existed some association between changes to mechanical threshold and disease severity, particularly in MBP animals, threshold trends persisted even following resolution of clinical paralysis. Overall, treatment with APL displayed a tendency to normalize pain thresholds, and hinder the development of mechanical pain hypersensitivity.

In MS, neuro-inflammatory lesions in the CNS produce significant somatosensory deficit, particularly in temperature discrimination, such as paradoxical heat sensations and altered heat/cold thresholds (52–54). In our experiments with male and female MBP animals, no differences in latency to thermal stimuli were observed, except in transience. Similarly, Olechowski et al. (21) reported no change in sensitivity to noxious heat, albeit using a different encephalitogenic peptide and animal model (21). In contrast, thermal hyperalgesia was observed in the tail and forepaws of male and female SJL mice, using a proteolipid protein from the myelin sheath as immunogenic source. These conflicting results underscore the high variability existent between differing animal models and encephalitogenic peptides. Consequently, we were not able to show any differences caused by APL co-immunization. It should be noted, however, that paralysis of the hind paws could have potentially confounded results, a concern shared by others (20, 21). Additional experimental setups that diminish the impact of paralysis on nociceptive testing, such as measuring vocalization response (55) or spontaneous pain (56) in animals, are encouraged.

As pain has only recently been recognized as a key functional disability of MS, a clear understanding of its pathogenesis is still absent. Several theories exist to explain this pain, including damage to somatosensory nerves (57), lesions in the CNS and spinal cord inflammation (17). However, a key factor is the dichotomous role of pro- and anti-inflammatory responses. Indeed, a recent study has shown that animals with EAE did not have altered expression of sensory neuropeptides, but possessed an influx of CD3^+^ T cells and increased astrocyte and microglia/macrophage reactivity in the superficial dorsal horn of the spinal cord, an area associated with pain processing (21). Additionally, a significant increase in the level of tumor necrosis factor (TNF) expression, a key pro-inflammatory cytokine, in the dorsal root ganglia of EAE animals was found at disease peak (58). Gene therapy with anti-inflammatory interleukin (IL)-10 resulted in prevention of the onset of allodynia in animals with EAE (50). Collectively, these findings suggest that pro-inflammation and gliosis are key mediators in the neuropathic pain behaviors associated with EAE.

The mechanisms underlying the analgesic effect of APL immunization in EAE-induced mechanical pain hypersensitivity are not known, but may include: reduced production of interferon-γ and TNF, pro-inflammatory cytokines that are critical in the pathogenesis of EAE (29); up-regulation of anti-inflammatory cytokines IL-4, IL-10, IL-13 and transforming growth factor-β (32); diverting immune responses from T_H_1 to T_H_2 (33); and mediating bystander suppression by the generation of regulatory T cells (59), which have been shown to suppress pain hypersensitivity in nerve-injured animals (60). In addition, APL immunization in EAE animals may have affected other pain mediators such as bradykinin, eicosanoids (prostaglandins and leukotrienes), adenosine-5′-triphosphate (ATP), histamine, chemokines (e.g., chemotactic cytokine ligand 2, fractalkine), neurotrophins (e.g., nerve growth factor, brain-derived neurotrophic factor), and reactive oxygen species to reduce mechanical pain hypersensitivity (16). Future studies will have to investigate the impact of APL treatment on immune modulation and inflammatory mediators associated with EAE pain.

While recent studies have mostly focused on individual single T cell clones in animal models (61), clinical trials have underlined the complexity of APLs. Despite APL’s effective suppression and reversal of EAE in rodents (29, 30), human trials reported conflicting results. In one phase II trial using [A^91^]MBP_83–99_, a decrease in the size of new MS lesions on MRI scans was observed in human subjects, but the trial was halted due to hypersensitivity reactions in 9% of patients (62). Crucially, there was no increase in disease exacerbation, although this did present in a similar phase II clinical trial (63). Thus, further clinical use of APLs is considered questionable. However, this avenue of research holds great promise, as the immune changes instigated by the APLs could induce 2–4.5 years of T_H_2-directed deviation in humans (64). Experimental trends show that clinical benefit and allergy mitigation is related to the correct dose of APL and route of administration, both of which necessitate further investigation (65).

Although our results highlight the restorative effect of APL on mechanical pain thresholds, further work is required to elucidate the mechanisms behind such changes. Challenges also remain in translating results from animal experiments into human therapies. Dosages need to be accurately titrated to maximize disease reduction while minimizing side effects, particularly T_H_2-induced hypersensitivity reactions. However, should this avenue of research yield promising results, it will herald a new field of immune deviation as a therapeutic option to neuropathic pain in MS and similar diseases.

## Conflict of Interest Statement

The authors declare that the research was conducted in the absence of any commercial or financial relationships that could be construed as a potential conflict of interest.
